# Anisotropy and Antagonism in the Coupling of Two Oscillators: Concepts and Applications for Between-Person Coordination

**DOI:** 10.3389/fpsyg.2016.01947

**Published:** 2016-12-21

**Authors:** Harjo J. de Poel

**Affiliations:** Center for Human Movement Sciences, University Medical Center Groningen, University of GroningenGroningen, Netherlands

**Keywords:** joint action, interpersonal dynamics, synchronization, social interaction, rhythmic coordination

## Abstract

Coupled oscillators provide a pertinent model approach to study between-person movement dynamics. While ample literature in this respect has considered the influence of external/environmental constraints and/or effects of a difference between the two agents' individual component dynamics (e.g., mismatch in natural frequency), recent studies also started to more directly consider the interaction *per-se*. The current perspective paper sets forth that while movement coordination dynamics has mainly been studied alongside a model in which the coupling is considered isotropic (i.e., symmetrical; both oscillators coupled to same degree) or strictly unidirectional (e.g., for moving to a given external rhythm), between-agent coupling involves a natural anisotropy: components influence each other bidirectionally to different degrees. Furthermore, recent research from different areas has considered so-called antagonistic or “competitive” coupling, which refers to the idea that one component is positively coupled to the other (attractive interaction), while the coupling in the other direction is negative (repulsive interaction). Although the latter would be rather tricky to address in within-person coordination, it does have strong applications and implications for between-person dynamics, for instance in the study of competitive interactions in sports situations (e.g., attacker-defender) and conflicting social (movement) interactions. The paper concludes by offering a conceptual framework and perspectives for future studies on the dynamic anisotropic nature of the interaction in between-person contexts.

## Introduction

Between-person coordination generally entails some form of functional cooperative synergy (Riley et al., 2011). Such collaborative coordination involves natural asymmetries, for example due to differences between the individual components. Indeed, ample literature examined coordinative performance as dependent on, for instance, a mismatch in natural frequency (i.e., “detuning;” e.g., Richardson et al., 2007) or movement amplitudes (e.g., de Poel et al., 2009; Fine, 2015). Broken symmetry also exists regarding the interaction itself (Treffner and Turvey, 1995; de Poel et al., 2007), yet this received considerably less attention in the coordination dynamics literature (see also Lagarde, 2013). The present perspective article therefore aims to highlight the study of such interactional asymmetries. Specifically, anisotropic (i.e., components influence each other bidirectionally to different degrees) and antagonistic coupling (i.e., one component attracts while the other repels) are deliberated in the context of dyadic between-person coordination. The paper concludes with a conceptual framework that may offer entry points for scientific engagements in this regard.

## Two coupled oscillators

The study of between-person coordination dynamics eminently draws from a pertinent model of coupled oscillators (Haken et al., 1985) known as the HKB-model (for a historic overview, see Schmidt and Fitzpatrick, 2016). While this model was originally developed for rhythmic bimanual coordination (i.e., within-person coordination), to date many studies have underwritten that between-person coordination abides by similar coordinative phenomena and principles (for reviews, see Schmidt and Richardson, 2008; Schmidt et al., 2011). Importantly, the component oscillators and coupling functions of the system are formulated such that it analytically constitutes a potential function that describes the attractor landscape of the collective behavior in terms of the phase difference (φ), capturing attractors at in-phase (φ = 0°), and antiphase behavior (φ = 180°) and their differential stability (Haken et al., 1985).

The general idea behind the coupled oscillator model is as follows. Two limit cycle (cf. self-sustaining) oscillators (reflected by subscript *i* = 1 or 2), each depicted by a second order differential equation are coupled following the general expression,

(1) $$\begin{array}{l} {\ddot{x}}_{1}+f(x_{1},{\dot{x}}_{1})=I_{12}\\ {\ddot{x}}_{2}+f(x_{2},{\dot{x}}_{2})=I_{21} \end{array}$$

in which *x*_*i*_, $\dot{x}$_*i*_, and ${\ddot{x}}_{i}$ reflect the position, velocity, and acceleration of the individual oscillators, respectively (because the present paper focuses on coupling, we assume identical oscillators), and *I*_12_ and *I*_21_ depict interaction functions that reflect the coupling between the two oscillators. Note that the couplings in *I*_12_ and *I*_21_ are a function of the difference between oscillator 1 and 2 in terms of their state variables (i.e., *x*_*i*_ and/or $\dot{x}$_*i*_), such as

(2) $$\begin{array}{l} I_{12}=\eta_{1}({\dot{x}}_{1}-{\dot{x}}_{2})\\ I_{21}=\eta_{2}({\dot{x}}_{2}-{\dot{x}}_{1}) \end{array}$$

(c.f., Astakhov et al., 2016), or as modeled by Haken et al. (1985) velocity- and position-dependent interaction of the form (see also Daffertshofer et al., 1999)

(3) $$\begin{array}{l} I_{12}=\eta_{1}\left(a_{1}+b_{1}{\left(x_{1}-x_{2}\right)}^{2}\right)({\dot{x}}_{1}-{\dot{x}}_{2})\\ I_{21}=\eta_{2}\left(a_{2}+b_{2}{\left(x_{2}-x_{1}\right)}^{2}\right)({\dot{x}}_{2}-{\dot{x}}_{1}) \end{array}$$

**Figure 1:**
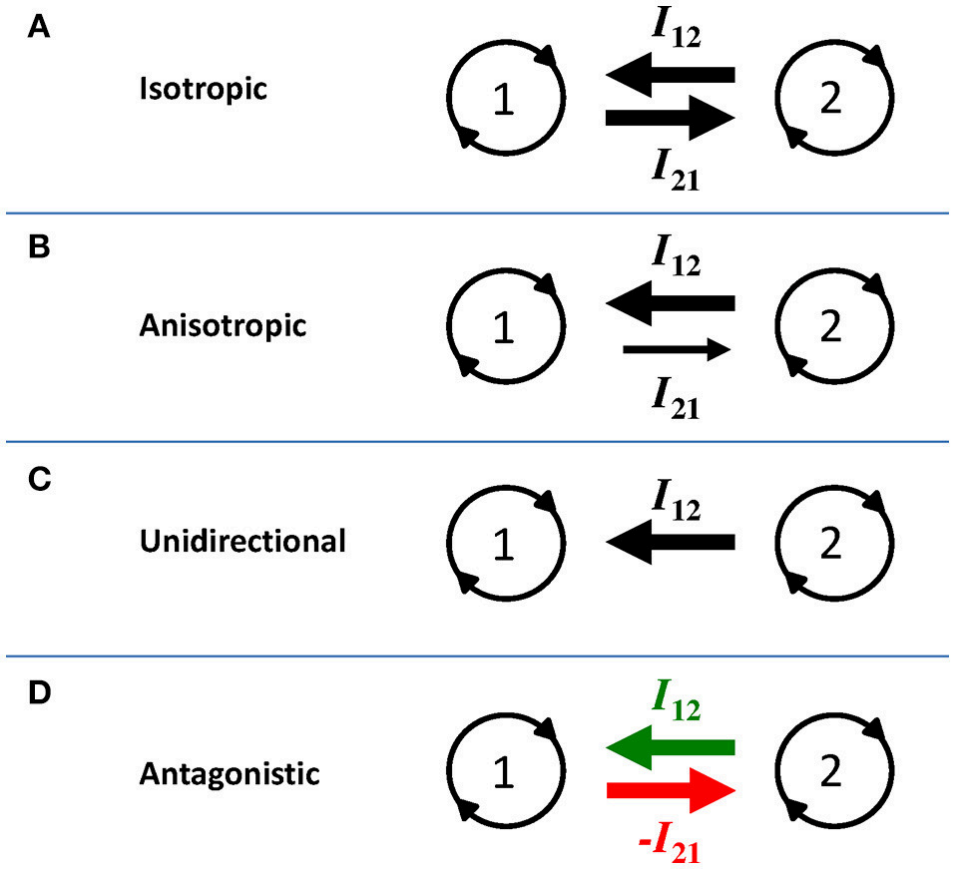
**Schematic illustration of four interaction scenarios (see main text for further explication)**. Oscillator components are represented by circles 1 and 2, and interactions between them (I_12_ and I_21_) represented by the arrows. The width of the arrows reflect the interaction strength in each direction, illustrating **(A)** isotropic coupling, **(B)** anisotropic coupling, **(C)** unidirectional coupling, and **(D)** antagonistic coupling. In **(D)**, attractive and repulsive coupling are emphasized by the green and red color of the arrow, respectively (see the online article for a colored version of this figure).

Regarding the purposes of this perspective article, we solely focus on general notions that can be derived and do not further consider the exact mathematical formulations. The first general notion from Equations (2) and (3) is that coupling coefficient η_*i*_ sizes the degree (or strength) of the coupling (for related modeling strategies in this context, see Varlet et al., 2012; Withagen et al., submitted). Obviously, when η_*i*_ = 0 there is no coupling whatsoever and the oscillators behave completely independently. Higher values of η_*i*_ imply stronger overall coupling and thus enhanced attractor stability at the relative phase level (Haken et al., 1985). When *I*_12_ and *I*_21_ are entirely identical the coupling is perfectly symmetric (such as assumed by Haken et al., 1985, who aimed at deriving a minimal model) meaning that both components influence one another to the same degree, as schematically illustrated in Figure 1A. However, while most previous studies on movement coordination adhered to this assumption (deliberately or not), the next paragraph will highlight that the coupling is anisotropic of nature and that such interactive asymmetry is substantial for understanding between-person coordination (see also Lagarde, 2013).

## Anisotropic coupling

Interaction between the components can be stronger in one direction than in the other, which implies an asymmetry in the strength of the coupling, hence anisotropic coupling (Peper et al., 2004; de Poel et al., 2007). From the preceding paragraph we can already see from Equations (2) and (3) that perfect isotropic coupling is an exceptional case: any other combination of coefficient values would yield *I*_12_ ≠ *I*_21_ and hence capture anisotropic coupling. This is schematically illustrated in Figure 1B. For bimanual coordination, anisotropic coupling has been related to hand dominance, which for instance yields a coordination pattern in which the dominant hand is slightly though systematically ahead of the non-dominant in terms of its movement phase (Treffner and Turvey, 1995; de Poel et al., 2007).

The anisotropy can obviously take different degrees. For instance, handedness-related anisotropy is less pronounced in left-handers than in right-handers (de Poel et al., 2007). Still, both limbs mutually influence each other: the interaction is clearly *bi*directional, be it with a certain degree of dependency-unevenness (Figure 1B). Increasing the coupling anisotropy toward the extreme form yields strict *uni*directional coupling, in which one component is influenced by the other, with no coupling whatsoever in reverse direction (e.g., when η_1_ ≠ 0 while η_2_ = 0, or vice versa). This situation essentially comes down to a forced oscillator (“master-slave;” see Figure 1C).

### “Leader-follower” dynamics

As in bimanual coordination (cf. de Poel et al., 2007), in dyadic coordination perfect symmetric interaction is the exception rather than the rule. To illustrate, a natural task such as crew rowing involves various sources to support interaction, amongst which a mechanical/haptic link via the boat that conveys more symmetrically, while the visual coupling is clearly asymmetric as the bow rower can see the movements of the stroke rower but not vice versa. The latter also draws in an explicit role division: the stroke rower sets the pace for the other rower(s) to adhere to (de Poel et al., 2016). Recently, researchers have started to examine such interactional directionalities in between-person settings, mainly in context of leader-follower relations (e.g., Konvalinka et al., 2010; Vesper and Richardson, 2014), such as in the context of a “mirror game” (Noy et al., 2011; Słowiński et al., 2016), of which some studies specifically pertained to (or referred to) a dynamic model of anisotropic/asymmetric coupling (Varlet et al., 2012; Meerhoff and de Poel, 2014; Fine, 2015; Richardson et al., 2015). Importantly, between-person coordination typically entails bidirectional “leader-follower” interaction rather than strict unidirectional “master-slave” dependency (e.g., Meerhoff and de Poel, 2014).

Regarding anisotropic interaction, some studies examined dyads in which agents differed in terms of their social competences or interactive skills (e.g., Schmidt et al., 1994; Varlet et al., 2012). Another way is to experimentally impose leader-follower conditions explicitly through instructions (e.g., Ducourant et al., 2005; Noy et al., 2011), or implicitly through reducing/precluding access to information in one direction (e.g., Meerhoff and de Poel, 2014; Reynolds and Osler, 2014). At the level of relative phase dynamics, anisotropic coupling predicts a specific lead-lag in the phase relation: the component that experiences the strongest coupling influence of the other is lagging (Treffner and Turvey, 1995; de Poel et al., 2007). In line, in between-persons experiments the “sighted” agent typically lags the “blind” (Meerhoff and de Poel, 2014; Reynolds and Osler, 2014).

When leader-follower situations are not explicitly dictated, isotropic coupling might be expected. It appears nothing is less true: implicit heuristic strategies seem to emerge that facilitate a “spontaneous” division in interactional roles of the dyad-members (Vesper et al., 2011; Richardson et al., 2015). In line, Meerhoff and de Poel (2014) found that even in the symmetric condition of their experiment, between-person coupling exhibited clear anisotropy for 70% of the examined pairs, indicating that there was almost always a “dominant interactor” within each pair. Such “intrinsic” leader-follower configuration may relate to the social dominance of one of the dyad-members (Schmidt et al., 1994). Furthermore, findings from interpersonal sway showed that in a situation where both dyad-members could see each other (i.e., symmetric visual coupling), cross-correlations of the sway patterns always involved a lag toward either side, whereas correlation was absent at lag zero (Reynolds and Osler, 2014). This also illustrates how in data analysis such asymmetries may be obscured due to averaging procedures.

Experiments on between-person coordination have mainly adopted tasks involving visual and/or auditory interface (for overviews, See Section Anisotropic Coupling of Repp and Su, 2013 and Schmidt and Richardson, 2008). Such perceptual coupling relies on an agent's sensitivity to, or ability to detect interaction-relevant information (Meerhoff and de Poel, 2014). Also, devoting less attention (Richardson et al., 2007) or simply closing the eyes (Oullier et al., 2008) would drastically diminish entrainment. In other words, anisotropic coupling may mainly reside in one oscillatory component being more susceptible to the interactional sources (“follower”) than the other (“leader”; de Poel et al., 2007), while an agent can also (whether or not intentionally) modulate the coupling influence inflicted on him/her (Withagen et al., submitted).

Together, these findings stress that between-person interaction is rarely symmetric and that typically one agent “leads the dance.” This notion is particularly interesting given that anistropically coupled oscillator dynamics may imply more stable coordinative attractors compared to the isotropic situation (provided overall coupling remains at same level, See Section Considerations and Perspectives and Treffner and Turvey, 1995; de Poel et al., 2007). In line, similar coupling asymmetries have been demonstrated to prosper performance of complementary joint action like a collision-avoidance task (Richardson et al., 2015). Together, this may provide incentives for why “leader-follower” collaboration may be beneficial over perfectly balanced interpersonal interaction.

## Antagonistic coupling

The preceding pertains to collaborative situations in which “leader” and “follower” cooperate toward a common task and/or to spontaneous interpersonal entrainment, in which two agents attract (to a certain, likely imbalanced degree) into one another's behavior. Most studies on movement coordination dynamics considered one (or both) of these scenarios of mutual attraction. Coupling influence can however also be repulsive or inhibitory (Kawahara, 1980; Kelso et al., 2009; Hong and Strogatz, 2011; Astakhov et al., 2016; Avitabile et al., 2016). Such repulsive interaction could for instance be modeled through setting the coupling coefficient η_*i*_ < 0 (Astakhov et al., 2016; note that Kelso et al., 2009, used a similar though slightly different modeling strategy). Hence, a high degree of repulsive coupling would reflect that the component is highly susceptible to coupling influence while inflicting repelling effect. Here, we specifically consider *antagonistic* coupling, which holds that one component attracts (positive coupling) while the other repels (negative coupling). It is principally a special case of anisotropic coupling with the inclusion of repulsive interaction, as schematically illustrated in Figure 1D.

In the context of between-person coordination antagonistic coupling is particularly relevant, as it may refer to conflictive social interactions (e.g., Liebovitch et al., 2008) or competitive opposition such as in sport (e.g., McGarry et al., 2002; Palut and Zanone, 2005). Note that the latter involves competitive attacker-defender rather than cooperative leader-follower interaction. As a simplified explication, in a truly competitive situation a defender aims to follow the attacker's movements (hence attraction to the attacker) while an attacker wants to behave diametrically opposed of what the defender does (hence repulsion from the defender). In other words, one agent looks to maintain the interactional balance while the other aims to break it. Interestingly, in a study of Kelso et al. (2009) movements of an avatar hand were real-time coupled to human hand movements through HKB-equation (i.e., according Equation 3), which allowed to examine “exotic” coupling parameter settings such as “reversed” coupling: The human was instructed to move in-phase with the avatar, while the avatar was programmed so as to achieve antiphase coordination, reflecting “conflict of intention.” Moreover, numerical simulations of HKB-coupled oscillators (viz. Equations 1 and 3) with repulsive coupling revealed that coordination was repelled from in-phase and antiphase, and instead converged toward 90° and/or −90° phase relations.

Also, Avitabile et al. (2016) recently demonstrated numerically that the HKB-model can indeed yield relative phase dynamics beyond in- and antiphase bistability, depending on the parameter regime adopted for the oscillator and coupling equations. In particular, they demonstrated that specific coefficient settings including a repulsive coupling can yield stable solutions shifting away from 0° and 180° toward 90° and −90° relative phase. Although they examined the model parameter settings in symmetric/isotropic fashion, these results may likely generalize toward antagonistic coupling, especially when broadening parameter ranges even further. This is an interesting route to explore in future studies. Furthermore, relevant for the present paper and according Frontiers Research Topic, Avitabile et al. (2016) also specifically discussed their modeling results vis-à-vis the potential interpretations regarding between-person dynamics.

Recently, we explored whether signs of antagonistic coupling could be observed in competitive dyadic interaction in sports (de Poel et al., 2014; see also McGarry and de Poel, 2016). We analyzed long baseline rallies taken from footage of official tennis matches at the highest competitive level (Association of Tennis Professionals tournaments). Relative phase was calculated from the lateral positions of both players on the tennis field (Palut and Zanone, 2005). Analysis of this data revealed high occurrence of in-phase and moreover even higher occurrence close to −90° and 90° relative phase. In hindsight, similar distributions appeared to be reported previously for squash data (McGarry, 2006) but were not interpreted vis-à-vis antagonistic coupling at the time. Further inspection of the tennis data showed that rallies consisted of periods in which the opponents appeared to balance their interaction (i.e., relative phase around 0°) and periods of clear competitive movement interaction (relative phase close to 90° and −90°). Notably, over the course of a rally the phase relation seemed to switch between these stages, likely reflecting that the odds change back and forth within rallies: sometimes one player dominated the rally (“attacker-defender”: 90°) whereas at other instances the other player dominated (“defender-attacker”: −90°), alternated with short periods of balance in which none of the opponents attempted to perturb the rally (“defender-defender”: 0°). A detailed report of these data will be provided elsewhere in a forthcoming paper.

## Considerations and perspectives

The preceding provides incentives for capturing and examining anisotropic coupling in the context of between-person coordination. Especially the idea of antagonistic coupling may offer novel insights for future analyses in this respect (cf. Kelso et al., 2009). To bolster such endeavors the paper concludes with a general schematic overview that captures the issues raised.

**Figure 2:**
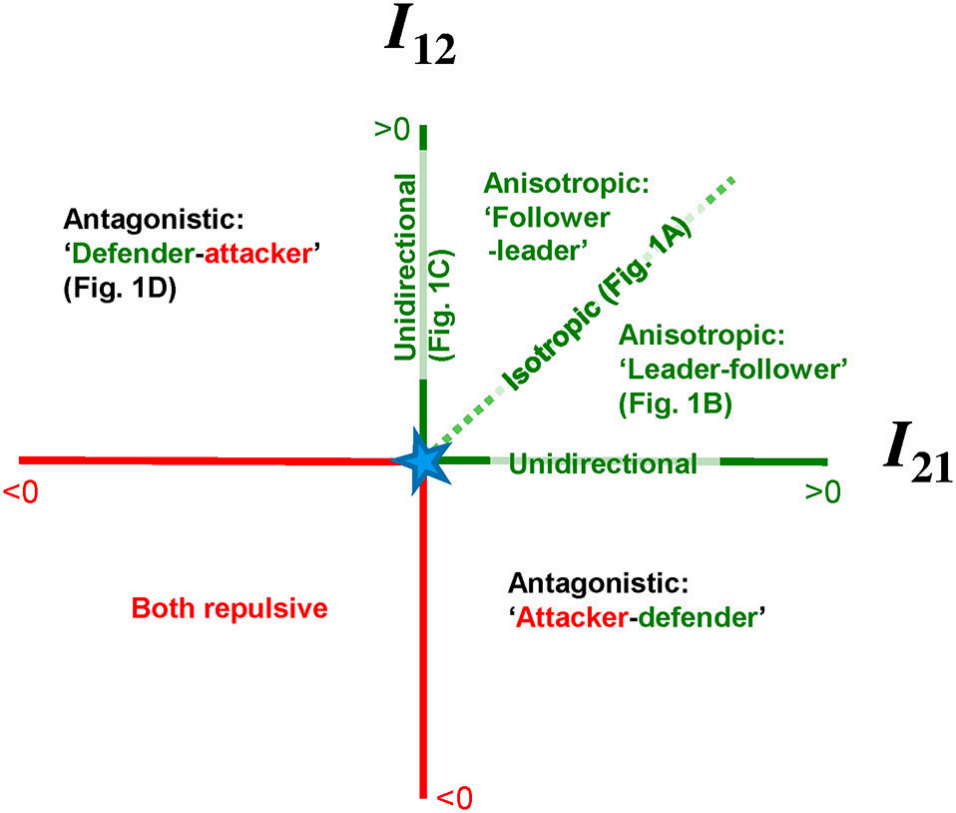
**Graphical illustration of coupling strengths in both directions in the I_**12**_–I_**21**_ coordinate frame**. For emphasis, issues regarding positive/attractive coupling are presented in green, while red reflects negative/repulsive coupling (see the online article for a colored version of this figure). For comparison, the four specific scenarios illustrated in Figure 1 are depicted at the according places. See main text for further explication.

Figure 2 graphically illustrates the coupling strength between the components in its proposed forms. The horizontal axis represents the degree of interaction inflicted on component 1 (*I*_12_) and on the vertical axis the coupling strength in the other direction is depicted (*I*_21_). Before we commence it is important to note that the interaction strength *I* and, thus, anisotropy therein is not solely defined by coupling coefficients, as it is a function of the individual oscillators (in terms of state variables *x*_*i*_ and/or $\dot{x}$_*i*_, see Equations 2–3). Indeed, in the HKB-model the coupling strength is strongly dependent on the movement amplitudes of the individual oscillators (Peper and Beek, 1999). Accordingly, in experiments with humans, amplitude disparity has been demonstrated to imply coupling anisotropy to a rather high degree (Peper et al., 2008). Hence, a difference between the individual component characteristics can involve an implicit coupling anisotropy, though the reverse is not necessarily true.

Returning to Figure 2, for any scenario the between-component coupling can be conceived as a point within the *I*_12_–*I*_21_ coordinate frame. Larger Euclidean distance from the origin indicates stronger interaction. The origin of the coordinate system (indicated by the star) evidently reflects the situation where there is no coupling (*I*_12_ = *I*_21_ = 0) and the diagonal in the second quadrant represents perfectly symmetric, isotropic coupling (*I*_12_ = *I*_21_ > 0) as discussed in Section Two Coupled Oscillators. The horizontal and vertical axis edging the second quadrant relate to unidirectional coupling (*I*_12_ = 0 while *I*_21_ > 0, or vice versa, See Section Anisotropic Coupling). The majority of previous studies on between-component movement coordination revolved their assumptions and/or inferences regarding coupling along this diagonal and/or these axes. The rest of the second quadrant reflects anisotropic coupling (Section “Leader-Follower” Dynamics). Note that while the anisotropy can be fairly large (i.e., further separated from the diagonal) the overall coupling can be stronger or weaker (i.e., further from/closer to the origin). This graphically illustrates that although stronger anisotropy may yield stronger coordinative attractor stability (Treffner and Turvey, 1995; de Poel et al., 2007) the latter primarily depends on *overall* interaction strength (cf. final paragraph of Section “Leader-Follower” Dynamics). Lastly, the other three quadrants depict repulsive coupling situations where at least one of the coupling influences *I* is negative. Specifically, antagonistic coupling (Section Antagonistic Coupling) is delineated in the first quadrant (*I*_12_ > 0 while *I*_21_ < 0; here the coupling influence acting on oscillator 2 is repulsive, hence agent 2 could be labeled as ‘attacker’) and fourth quadrant (*I*_12_ < 0 while *I*_21_ > 0; here agent 1 is the ‘attacker’). The third quadrant considers a situation in which interaction is repulsive in both directions, which was beyond the scope of the present paper and remains to be further investigated.

In sum, the present study offered a brief overview for the perspective that (1) between-person coupling is typically anisotropic, and (2) can also take repulsive/antagonistic shapes. The presented conceptual framework may provide incentives for further study of coupled oscillator models (e.g., in terms of analytical and/or numerical examination of anisotropic and antagonistic coupling settings) and related empirical examinations. For instance, the antagonistic experimental design of Kelso et al. (2009) may be translated to an agent-agent (rather than agent-avatar) situation, where one participant gets the instruction to move in-phase with his/her partner, while the other gets an antiphase instruction. Notably in this context, compared to within-person coupling, between-person coupling arguably allows for (empirical examination of) a larger variety of coupling settings (see also Avitabile et al., 2016), like antagonistic coupling.

## Author contributions

The author confirms being the sole contributor of this work and approved it for publication.

### Conflict of interest statement

The author declares that the research was conducted in the absence of any commercial or financial relationships that could be construed as a potential conflict of interest.
